# Real-world use of complement inhibitors for haemolytic uraemic syndrome: an analysis of the European Rare Kidney Disease Registry cohort

**DOI:** 10.1016/j.eclinm.2025.103159

**Published:** 2025-03-27

**Authors:** Aleksandra Vujović, Anne-Laure Sellier-Leclerc, Maria Cristina Mancuso, Olivia Boyer, Atif Awan, Antonio Gargiulo, Sebastian Loos, Marc Fila, Augustina Jankauskiene, Gema Ariceta, Nele Kanzelmeyer, Enrico Vidal, Maria Van Dyck, Tanja Kersnik Levart, Naděžda Šimánková, Stephane Decramer, Jonas Hofstetter, Marina Vivarelli, Savino Sciascia, Nicole C.A.J. van de Kar, Franz Schaefer, ERKNet TMA Working Group, Nicole C.A.J. van de Kar, Marina Vivarelli, Savino Sciascia, David Kavanagh, René Andersen, Mia Faerch, Soren Rittig, Miquel Blasco, Pedro Arango Sancho, Alvaro Madrid, Gema Ariceta, Loreto Gesualdo, Camillo Carrara, Piero Ruggenenti, Kai-Uwe Eckardt, Jan Halbritter, Dominik Müller, Adrian Schreiber, Evelyn Seelow, Yahsou Delmas, Jerome Harambat, Brigitte Llanas, Nathalie Godefroid, Eric Goffin, Johann Morelle, Adrian Catalin Lungu, George Reusz, Péter Sallay, Attila Szabo, Kalman Tory, Jan Ulrich Becker, Kathrin Burgmaier, Volker Burst, Sandra Habbig, Max Liebau, Roman-Ulrich Mueller, Lutz Weber, Mette Damholt, Anne-Lise Kamper, Karl Emil Nelveg-Kristensen, Hanne Nørgaard, Ida Maria Schmidt, Soeren Soerensen, Wladimir Szpirt, Agnieszka Jaskólska, Monika Miklaszewska, Anna Moczulska, Elżbieta Szczęsny-Choruz, Katarzyna Zachwieja, Peter Conlon, Atif Awan, Michael Wiesener, Anja Büscher, Rainer Büscher, Lars Pape, Francesca Becherucci, Paola Romagnani, Ann Raes, Thomas Renson, Evelien Snauwaert, Johan Vande Walle, Jill Vanmassenhove, Mark Eijgelsheim, Casper Franssen, Coen Stegeman, Florian Grahammer, Thomas Henne, Tobias Huber, Christian Krebs, Sebastian Loos, Anne Mühlig, Jun Oh, Ulf Panzer, Raphael Schild, Jessica Kaufeld, Stefanie Haeberle, Franz Schaefer, Tanja Wlodkowski, Juuso Tainio, Elisa Ylinen, Obbo Bredewold, Wieneke Michels, Dorien Peters, Antonius Rabelink, Arghya Ray, Joris Rotmans, Siebe Spijker, Y.K. Onno Teng, Kathleen Claes, Noel Knops, Tanja Kersnik Levart, Anamarija Meglič, Gregor Novljan, Agnieszka Gach, Monika Pawlak-Bratkowska, Małgorzata Stańczyk, Marcin Tkaczyk, Pierre Cochat, Anne-Laure Sellier-Leclerc, Teresa Cavero, Eduardo Guiterrez, Joaquin Martinez, Enrique Morales, Hernando Trujillo, Gianluigi Ardissino, Valentina Capone, Sara Testa, Denis Morin, Martin Konrad, Ilaria Luongo, Gabriele Malgieri, Luigi Annicchiarico Petruzzelli, Wilbert van der Meijden, Jack Wetzels, Mattia Parolin, Enrico Vidal, Thérésa Kwon, Olivia Boyer, Aude Servais, Laurent Mesnard, Alena Parikova, Silvie Rajnochova-Bloudickova, Janka Slatinska, Ondrej Viklicky, Martin Bezdicka, Nadezda Simankova, Dana Thomasova, Jakub Zieg, Francesco Emma, Rocco Baccaro, Giuseppe Grandaliano, Alessandro Naticchia, Francesco Pesce, Margherita Baldassarri, Andrea Guarnieri, Anna Maria Pinto, Alessandra Renieri, Stephane Decramer, Stanislas Faguer, David Ribes, Thomas Simon, Stephanie Tellier, Daniel Gale, Christoph Licht, Michal Malina, Candice Roufosse, Neil Sheerin, Susana Carvajal Arjona, Renée de Wildt, Uwe Korst, Christiane Mockenhaupt, Francisco Monfort, Mireya Vicenta Rios Carratala

**Affiliations:** a Division of Paediatric Nephrology, University Children's Hospital, University Medical Centre Ljubljana, Ljubljana, Slovenia; b Faculty of Medicine, University of Ljubljana, Ljubljana, Slovenia; c Service de Néphrologie Pédiatriques, Centre de Référence Des Maladies Rénales Rares Néphrogones Filières Maladies Rares ORKID et ERKNet, Hospices Civils de Lyon, Bron, Lyon, France; d Division of Paediatric Nephrology, Dialysis and Transplantation, Fondazione IRCCS Ca' Granda, Ospedale Maggiore Policlinico, Milan, Italy; e Paediatric Nephrology, Necker Enfants Malades Hospital, MARHEA Reference Centre, Imagine Institute, Université Paris Cité, France; f Division of Paediatric Nephrology, Children's Health Ireland at Temple Street, Temple Street, Ireland; g Division of Paediatric Nephrology and Dialysis, Bambino Gesù Children's Hospital, IRCCS, Rome, Italy; h Division of Paediatric Nephrology, University Medical Centre Hamburg-Eppendorf, Hamburg, Germany; i Division of Paediatric Nephrology and Dialysis, CHU Arnaud de Villeneuve, Centre De Référence Des Maladies Rénales Rares du Sud-Ouest (SORARE), Montpellier, France; j Paediatric Centre, Institute of Clinical Medicine, Vilnius University, Vilnius, Lithuania; k Paediatric Nephrology, Vall d'Hebron Hospital, Autonoma University of Barcelona, Barcelona, Spain; l Department of Paediatric Kidney, Liver and Metabolic Diseases, Hannover Medical School, Children's Hospital, Hannover, Germany; m Division of Paediatric Nephrology, Department of Women's and Child's Health, University Hospital of Padova, Padua, Italy; n Division of Paediatric Nephrology, University Hospitals Leuven, Leuven, Belgium; o Department of Paediatrics, Second Faculty of Medicine, Charles University and Motol University Hospital, Prague, Czech Republic; p Department of Paediatric Internal Medicine, Rheumatology and Nephrology, Toulouse University Hospital, Toulouse, France; q Centre De Référence Des Maladies Rénales Rares du Sud-Ouest (SORARE), Toulouse University Hospital, Toulouse, France; r National Institute of Health and Medical Research (INSERM), UMR 1297, Institute of Cardiovascular and Metabolic Disease, Toulouse, France; s Division of Paediatric Nephrology, Department of Paediatrics, University of Heidelberg, Heidelberg, Germany; t Division of Nephrology, University of Torino-Ospedale HUB Torino Nord, Turin, Italy; u Division of Paediatric Nephrology, Radboud University Nijmegen Medical Centre, Nijmegen, the Netherlands

**Keywords:** Haemolytic uraemic syndrome, Complement inhibitor, Treatment discontinuation, Post-withdrawal relapse, European Rare Kidney Disease Registry

## Abstract

**Background:**

Although terminal complement inhibitors transformed the prognosis of atypical haemolytic uraemic syndrome (aHUS) from dismal to favourable, treatment approaches vary due to the intermittent disease nature and high costs. Occasionally, complement inhibition is applied in infectious (i)HUS. We aimed to examine real-world C5 inhibitor use and its impact on patient outcomes.

**Methods:**

This retrospective cohort study used longitudinal data from the European Rare Kidney Disease Registry, collected from 76 nephrology centres across 24 European countries between January 1, 2019 and January 31, 2024. Eligible patients had aHUS or iHUS with onset after January 1, 2011, and/or documented C5 inhibitor use. Exclusions included complement-unrelated HUS, post-transplant HUS, and prophylactic C5 inhibitor use around kidney transplantation. Data, derived from medical records and focused queries, were used to assess C5 inhibitor duration, via time-to-event analysis, and kidney function based on annual creatinine levels.

**Findings:**

A total of 238 aHUS and 472 patients with iHUS were included in the analysis. C5 inhibition was applied in 76.5% of aHUS and 18.4% of iHUS, with major utilisation differences between countries (p < 0.0001) and less common use in female patients with aHUS (p = 0.0022). Median (interquartile range) treatment duration was 16.1 (3.6–41.2) months in aHUS and 9 (7–32) days in iHUS. After five years, 56% of genetic, 28% of anti-complement factor H (anti-CFH) antibody-mediated, and 23% of aHUS cases with no identified cause remained on treatment. The long-term (>7 years) risk of treatment resumption was 35% in genetic, 15% in aHUS of no identified cause, and 0% in anti-CFH antibody-mediated aHUS. Post-withdrawal aHUS relapses were mostly mild and did not lead to permanent kidney function impairment, ultimately leading to long-term treatment withdrawal in 92.5% of discontinued cases.

**Interpretation:**

Currently, C5 inhibitors are administered in three-quarters of newly diagnosed patients with aHUS in Europe, with varied utilisation and discontinuation practices. Treatment withdrawal is common and safe, although relapses may occur, particularly in genetic aHUS. However, baseline disease severity, selective use in expert centres, and indication bias affect outcome comparability. Findings must be considered in the context of patient-specific factors and disease severity at the time of treatment decisions.

**Funding:**

This research was supported by the 10.13039/501100021081European Reference Network for Rare Kidney Diseases, funded by the 10.13039/100011105European Union within the framework of the “EU4Health Programme 2021–2027”.

**Research in context**

**Evidence before this study**

A comprehensive PubMed search of studies and references listed in the identified papers, published up to August 25, 2023, was conducted using search terms such as C5 inhibitor, eculizumab, ravulizumab, atypical haemolytic uraemic syndrome (aHUS), and discontinuation of C5 inhibitors, with search terms found in abstracts, titles, or MeSH headings. Previous studies have indicated the efficacy of C5 inhibitors in managing aHUS and provided insights into treatment discontinuation and relapse risk. Despite these findings, real-world data on long-term outcomes, particularly regarding the safety of treatment discontinuation and post-withdrawal relapses, remain limited.

**Added value of this study**

This is the largest real-world analysis of clinical practices in aHUS and iHUS since introducing C5 inhibitors, providing evidence on the long-term safety of treatment discontinuation. Our findings suggest that relapses after withdrawal are generally mild, do not cause permanent kidney damage, and are effectively managed with early therapy resumption.

**Implications of all the available evidence**

These findings suggest that while C5 inhibitors are effective in managing aHUS, treatment discontinuation is feasible and safe in a significant number of patients. This supports the potential for personalised, cost-effective treatment strategies based on genetic and clinical risk factors. However, as an observational study, differences in baseline disease severity, selective use in expert centres, and indication bias may influence the interpretation of outcomes, emphasising the need for further research to optimise discontinuation strategies and improve post-withdrawal relapse management.

## Introduction

Over the past decade, the introduction of terminal complement inhibitors has profoundly transformed the management and outcome of atypical haemolytic uraemic syndrome (aHUS), an ultra-rare multi-systemic disease characterised by severe and/or life-threatening acute kidney injury, thrombocytopaenia, and non-immune microangiopathic haemolytic anaemia. In 2011, eculizumab was approved for the treatment of aHUS in the United States (US) and the European Union (EU), followed by the approval of ravulizumab in the US and the EU in 2018 and 2019, respectively. Complement inhibitors have considerably improved the historically dismal prognosis of aHUS, with significant enhancements in patient and kidney survival as well as quality of life.^1^^,^^2^

Due to the episodic nature of thrombotic microangiopathy (TMA) events and the exorbitantly high costs of the drugs, treatment discontinuation strategies have been proposed and applied in the recent past.^3^ These strategies differ in risk stratification, timing of discontinuation, and post-withdrawal management of relapsed patients, creating a need for real-world evidence on treatment practices and outcomes in this ultra-rare condition.

Another aspect of interest is the use of eculizumab in patients with infectious forms of HUS. Anecdotal experience in patients with HUS caused by Shiga-toxin producing *Escherichia coli* (STEC) with systemic complement activation and extrarenal disease manifestations suggested a beneficial effect of C5 inhibition.^4^ A recent randomised, placebo-controlled trial in children with STEC HUS excluding cases with severe presentation did not show efficacy of eculizumab during the acute phase but suggested a reduction of renal sequelae at one-year follow-up.^5^ In contrast, a systematic review of medium- and long-term outcomes of eculizumab for severe STEC HUS found no significant positive effect; however, due to the high risk of bias in the studies reviewed, the potential effectiveness of eculizumab may have been underestimated.^6^

Since 2019 the European Rare Kidney Disease Registry (ERKReg) has collected longitudinal real-world information on the management of patients with rare and ultra-rare kidney disorders including atypical and infectious HUS.^7^ Here, we interrogated the ERKReg database to identify the patterns of use of complement inhibitors for these conditions at European expert centres and analyse patient outcomes on and off treatment.

## Methods

### Cohort selection

This retrospective cohort study used longitudinal data collected on HUS patients between January 1, 2019 and January 31, 2024, in ERKReg. ERKReg, an initiative of the European Reference Network for Rare Kidney Diseases (ERKNet), collects real-world data at currently 76 specialised adult and paediatric nephrology centres across 24 countries. Each centre obtained approval from its local ethics committee. Written informed consent was secured from patients or their legal representatives for inclusion in the ERKReg. Data extraction for this study encompassed the precise HUS diagnosis (by Orphacode), demographic information, genetic and other diagnostic findings, and treatment modalities (plasma exchange, acute and chronic dialysis, transplantation), as well as kidney function data recorded at the point of registry enrolment and assessed through annual updates of serum creatinine levels. Race data were collected as an investigator-assigned variable, where investigators classified patients into one of the predefined categories: European, East Asian, Hispanic, Indian, Arabic, African/African American, Other, or Unknown. Additional information on the causes of primary non-use of C5 inhibitors, as well as the clinical and biochemical courses of post-discontinuation TMA episodes in patients with aHUS was collected through focused queries. All patients with a diagnosis of HUS were included in the analysis if complement inhibitor treatment information was available and disease onset was after 2011, the year of eculizumab approval in the EU, or before 2011 if they had received eculizumab within a trial. All analyses were performed using available data, without imputation or estimation of missing values. The overall missing rate across all key variables used in the statistical analysis was 2.99%. This study adheres to Strengthening the Reporting of Observational Studies in Epidemiology (STROBE) guidelines.

### Statistics

Descriptive statistics of continuous variables were presented as means and standard deviations (SD) for variables with normal distributions, or as medians and interquartile ranges (IQR) for variables with non-normal distributions. The D'Agostino-Pearson test was used to determine if the distributional normality assumption had to be rejected for continuous variables. Categorical variables were summarised by counts and percentages. For testing differences between two groups on continuous measures, Welch's independent samples t-test was used as a default method but replaced by a Mann–Whitney U test when a non-normal distribution had been detected for at least one of the compared groups. For comparisons of continuous variables among more than two groups, one-way analysis of variance (ANOVA) was employed as a default method but replaced by a non-parametric Kruskal–Wallis test if at least one group exhibited a non-normal distribution, or when Levene's test results indicated unequal variances between groups. Fisher's exact test was used to test for associations between categorical measures. Kaplan–Meier survival analysis was employed to characterise the duration of complement inhibitor use or post-withdrawal non-use in subgroups of interest. Multivariable Cox regression was performed to identify factors affecting the likelihood of treatment discontinuation. Formal statistical tests and graphical diagnostic plots based on derived scaled Schoenfeld residuals and Martingale residuals were utilised to validate the Cox regression's proportional hazards assumption for all explanatory covariates across time t and the linearity assumption of the modelled log event hazard with the continuous explanatory covariate age at diagnosis.

### Role of the funding source

The funding body had no role in the study design, data collection, data analysis, data interpretation, or in the writing of the report or the decision to submit this paper for publication. AV, FS, and JH accessed and verified the underlying data and held final responsibility for the decision to submit the manuscript.

## Results

### Cohort composition

Out of a total population of 942 registered patients diagnosed with HUS, the following patients were excluded from the analysis: 35 due to missing information on complement inhibitor, 167 diagnosed with aHUS before 2011 who had not received complement inhibitor treatment, 13 with complement-unrelated forms of aHUS (seven with Cobalamin C deficiency and six with Diacylglycerol Kinase Epsilon (DGKE) nephropathy), three in whom HUS first manifested after kidney transplantation, and two infectious (i)HUS cases with inconsistent data. Additionally, 12 patients receiving prophylactic complement inhibitor therapy around kidney transplantation were also excluded. The cohort included 710 patients, of whom 238 had aHUS and 472 had iHUS. Patients with aHUS were divided into three groups based on aetiology: anti-complement factor H (anti-CFH) antibody-mediated (n = 48), genetic (n = 85), and no identified cause (n = 105). In the latter group, genetic testing was not performed in 60 patients, 34 had negative test results, seven had variants of uncertain significance (including five in complement factor H-related genes 3 and 1 (CFHR3/CFHR1), one in complement factor I (CFI) and one in CD46 molecule (CD46)), and results were pending for four patients. The iHUS cohort consisted of 472 patients, classified into two categories: 456 with STEC HUS and 16 with *Streptococcus pneumoniae*-associated HUS (Pneumococcal HUS).

#### Overall characteristics

The study followed patients who were reported to the registry by 41 specialised units (36 paediatric and 11 adult nephrology) in 18 European countries. Of the 710 patients included, the majority (582, 82%) were classified as European, while 28 (4%) were Arabic, 11 (2%) African or African American, 10 (1%) Hispanic, 2 (<1%) Indian, 1 (<1%) East Asian, 11 (2%) classified as Other, and 65 (9%) had an unknown racial background (not shown). Global patient characteristics are presented in Table 1. The majority of HUS cases had childhood onset, with adult-onset observed in 11.1% (23/208) of aHUS and 0.2% (1/435) of iHUS cases (p < 0.0001). Patients with aHUS were generally older at disease onset (p < 0.0001) and were followed up longer than patients with iHUS (p < 0.0001). Patients with genetic aHUS were followed up longer than patients with no cause identified aHUS (p = 0.0044).

**Table 1:** Patient characteristics of atypical haemolytic uraemic syndrome (aHUS) and infectious haemolytic uraemic syndrome (iHUS) cohorts.

	Comparisons by disease aetiology for patients with aHUS only				Comparisons by disease type		
	Genetic	Anti-CFH antibody	No cause identified	p value	All aHUS	Infectious HUS	p value
N	85	48	105		238	472	
Sex				0.85			0.095
N (%) female	39/85 (45.9)	24/48 (50.0)	47/105 (44.8)		110/238 (46.3)	250/472 (53.0)	
N (%) male	46/85 (54.1)	24/48 (50.0)	58/105 (55.2)		128/238 (53.7)	222/472 (47.0)	
N (%) adult-onset	9/74 (12.2)	1/44 (2.3)	13/90 (14.4)	0.078	23/208 (11.1)	1/435 (0.2)	<0.0001
Age at disease onset (y)	4.3 (1.0; 10.2)	6.0 (4.3; 8.3)	5.0 (1.3; 13.0)	0.57	4.8 (1.3; 9.2)	2.3 (1.3; 4.7)	<0.0001
Duration of follow-up (y)	5.7 (2.8; 8.4)	5.1 (2.9; 7.2)	3.2 (1.0; 7.3)	0.0044	5.0 (1.3; 9.4)	2.3 (1.3; 5.0)	<0.0001
N (%) receiving C5 inhibitor at any time	69/85 (81.2)	40/48 (83.3)	73/105 (69.5)	0.084	182/238 (76.5)	87/472 (18.4)	<0.0001

Legend: Abbreviations: aHUS—atypical haemolytic uraemic syndrome; anti-CFH antibody—anti-complement factor H antibody-mediated aHUS; N—number of patients; %—percentage; y—year.

Data given as N (% of patients with non-missing data) for categorical variables and as median (interquartile range: 25th–75th percentiles) for continuous variables.

Data availability for genetic aHUS: sex 85/85, adult-onset of disease 74/85, age at disease onset 74/85, duration of follow-up 82/85, and receipt of C5 inhibitor therapy at any time 85/85.

Data availability for anti-CFH antibody mediated aHUS: sex 48/48, adult-onset of disease 44/48, age at disease onset 44/48, duration of follow-up 46/48, and receipt of C5 inhibitor therapy at any time 48/48.

Data availability for no cause identified aHUS: sex 105/105, adult-onset of disease 90/105, age at disease onset 90/105, duration of follow-up 100/105, and receipt of C5 inhibitor therapy at any time 105/105.

Data availability for all aHUS: sex 238/238, adult-onset of disease 208/238, age at disease onset 208/238, duration of follow-up 228/238, and receipt of C5 inhibitor therapy at any time 238/238.

Data availability for infectious HUS: sex 472/472, adult-onset of disease 435/472, age at disease onset 435/472, duration of follow-up 453/472, and receipt of C5 inhibitor therapy at any time 472/472.

#### C5 inhibitor treatment in aHUS

Among the 238 patients in the registry who had aHUS, 182 (76.5%) received C5 inhibitor treatment, without a difference between the aetiological subgroups (p = 0.084; Table 1).

Reasons for not applying C5 inhibitor treatment (provided for 28 of 56 patients) included mild clinical presentation characterised by the absence of acute kidney injury requiring dialysis (n = 13), no relapse after plasma exchange (n = 5), delayed diagnosis (n = 4), delays in national drug approval (n = 3), advanced chronic kidney disease (CKD) at presentation (n = 2), and financial constraints (n = 1) (not shown). Eleven of the 13 patients with mild clinical presentation were children. In five of these patients, a genetic cause was identified (2 variants in complement C3 (C3) and one each in CFH, CFI and CD46), while two patients had negative genetic results and in six patients no genetic testing was performed (not shown). At the last observation, 11 of these patients were classified as CKD stage 1 and two had progressed to CKD stage 3 (not shown).

Notably, female patients were less likely to receive C5 inhibitor treatment than males (p = 0.0022; Table 2). However, an analysis of male and female cohorts who never received C5 inhibitors revealed that the cohorts were comparable in terms of genetic testing results (p = 0.16), diagnosis distribution (p = 0.45), and CKD stages (p = 0.33), respectively (not shown). A significant difference in treatment continuity was observed across aetiological groups, with sustained treatment being more frequent in patients with a genetic cause and less common in those without an identified cause (p = 0.0019; Table 2). Eculizumab was used as initial therapy in 178 patients, while four patients were started on ravulizumab. Twenty-five (14.0%) patients were switched from eculizumab to ravulizumab and five (2.8%) to crovalimab after median (IQR) treatment times of 3.5 (1.4–5.9) and 4.5 (1.8–6.0) years, respectively.

**Table 2:** Characteristics of patients with atypical haemolytic uraemic syndrome (aHUS) with sustained, discontinued, and no C5 inhibitor treatment.

	Comparison by C5 inhibitor treatment			Comparisons by continuity of C5 inhibitor treatment		
	Never treated	Treated	p value	Sustained treatment	Treatment discontinued	p value
N	56	182		73	109	
N (%) female	36/56 (64.3)	74/182 (40.7)	0.0022	28/73 (38.4)	46/109 (42.2)	0.65
N (%) adult-onset	3/40 (5.4)	20/168 (11.9)	0.58	8/69 (11.6)	12/99 (12.1)	0.99
% disease onset <2015/2015–2019/≥2019	27.5/22.5/50	28.6/35.1/36.3	0.22	29.0/27.5/43.5	27.8/42.3/29.9	0.087
% genetic/anti-CFH ab/no identified cause	28.6/14.3/57.1	37.9/22.0/40.1	0.084	53.4/16.5/30.1	27.5/25.7/46.8	0.0019

Legend: Abbreviations: N—number of patients; %—percentage; anti-CFH ab—anti-complement factor H antibody-mediated aHUS.

Data given as N (% of patients with non-missing data).

Data availability for Never treated: sex 56/56, adult-onset of disease 40/56, year of disease onset 40/56, and diagnosis 56/56.

Data availability for Treated: sex 182/182, adult-onset of disease 168/182, year of disease onset 168/182, and diagnosis 182/182.

Data availability for Sustained Treatment: sex 73/73, adult-onset of disease 69/73, year of disease onset 69/73, and diagnosis 73/73.

Data availability for Treatment discontinued: sex 109/109, adult-onset of disease 99/109, year of disease onset 99/109, and diagnosis 109/109.

#### Treatment discontinuation

**Fig. 1:**
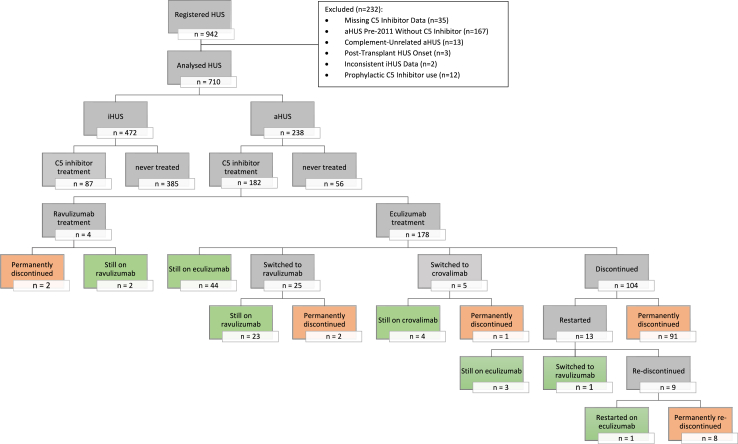
Disposition of C5 inhibitor treatment in haemolytic uraemic syndrome (HUS) cohort. This figure shows the distribution of patients based on their treatment status. Orange represents permanent treatment discontinuation, and green indicates sustained treatment. Abbreviations: HUS—haemolytic uraemic syndrome; iHUS—infectious haemolytic uraemic syndrome; aHUS—atypical haemolytic uraemic syndrome.

**Fig. 2:**
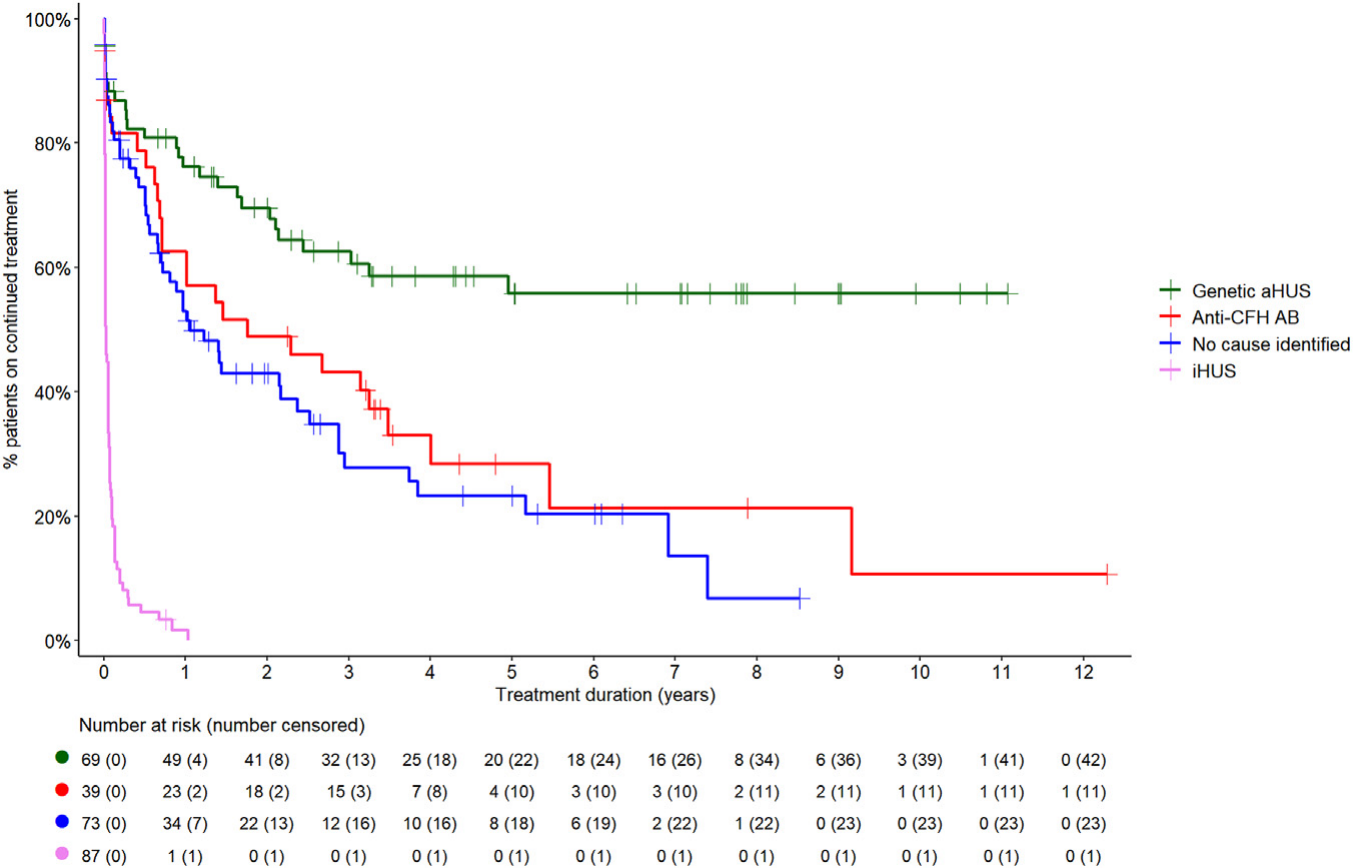
Duration of C5 inhibitor treatment by haemolytic uraemic syndrome (HUS) aetiology. This figure illustrates the duration of C5 inhibitor treatment across different HUS aetiologies. Green shows genetic aHUS, red indicates anti-CFH antibody-mediated aHUS, blue represents cases with no identified cause, and pink represents iHUS cases. Abbreviations: %—percentage; aHUS—atypical haemolytic uraemic syndrome; anti-CFH ab—anti-complement factor H antibody-mediated aHUS; iHUS—infectious haemolytic uraemic syndrome. Data availability for the duration of C5 inhibitor treatment: genetic aHUS 69/69, anti-CFH antibody-mediated aHUS 39/40, aHUS with no identified cause 73/73, and iHUS 87/87.

**Fig. 3:**
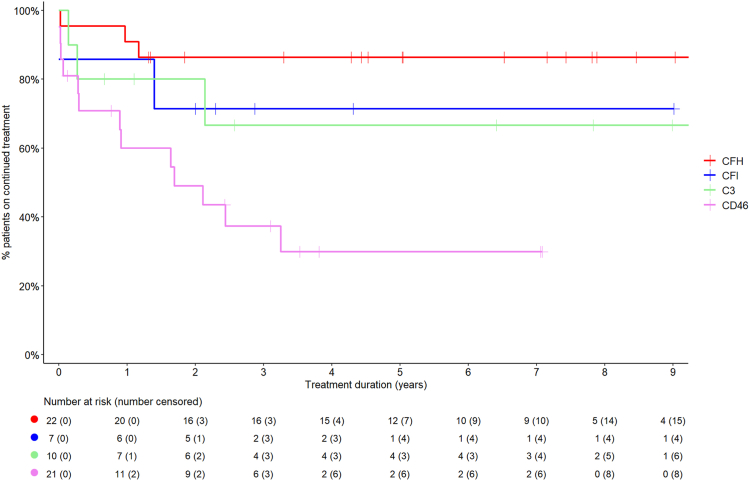
Duration of C5 inhibitor treatment in patients with genetic atypical haemolytic uraemic syndrome (aHUS) by affected gene. This figure depicts the duration of C5 inhibitor treatment in patients with genetic aHUS categorised by their affected genes. Red indicates CFH mutations, blue CFI mutations, green C3 mutations, and pink CD46 mutations. Abbreviations: %—percentage; CFH—complement factor H; CFI—complement factor I; C3—complement component 3; CD46—CD46 molecule. Data availability for the duration of C5 inhibitor treatment in genetic aHUS: CFH 22/23, CFI 7/7, C3 10/10, and CD46 21/21.

Among the 182 patients with aHUS treated with C5 inhibitors, treatment was discontinued in 109 (104 eculizumab, 4 ravulizumab, 1 crovalimab), as shown in Fig. 1. The median (IQR) duration of initial C5 inhibitor treatment (including patients who switched from eculizumab to ravulizumab or crovalimab) was 16.1 (3.6–41.2) months. Treatment duration differed significantly by disease aetiology (p < 0.0001; Fig. 2). C5 inhibition was sustained longest in patients with genetic forms of aHUS, 56% of whom were still on treatment after 5–11 years of observation. Patients with anti-CFH antibody-mediated aHUS were treated shorter, with 51% discontinuation at 22 months and 89% at the last observation. Among patients with aHUS without an identified cause, treatment was withdrawn within 13 months in 50% and within five years in 77% of cases. Among patients with genetic forms of aHUS, 86% of those with CFH mutations, 83% with CFI mutations, and 67% with C3 mutations were maintained on long-term C5 inhibition, whereas only 35% of patients with CD46 mutations were treated for longer than three years (p = 0.0040; Fig. 3). Among 21 patients with aHUS who progressed to kidney failure, nine had been started on C5 inhibition within three months of kidney failure onset. This group included one patient with CKD 5, seven patients who remained dialysis-dependent at the last follow-up, and one who underwent kidney transplantation. Among the nine patients who started C5 inhibition, three discontinued treatment when kidney failure persisted for three months. Furthermore, 12 patients with aHUS treated with C5 inhibitor showed late progression to permanent kidney failure after a median (IQR) of 3.0 (2.2–5.6) years on treatment. Among these, one patient was in CKD 5, one was on dialysis and ten had received a kidney transplant at the last observation. C5 inhibition was discontinued in six of these 12 patients: in four cases before permanent kidney failure occurred, in one case after kidney transplantation, and in one case temporarily around the time of transplantation but treatment was resumed later.

**Fig. 4:**
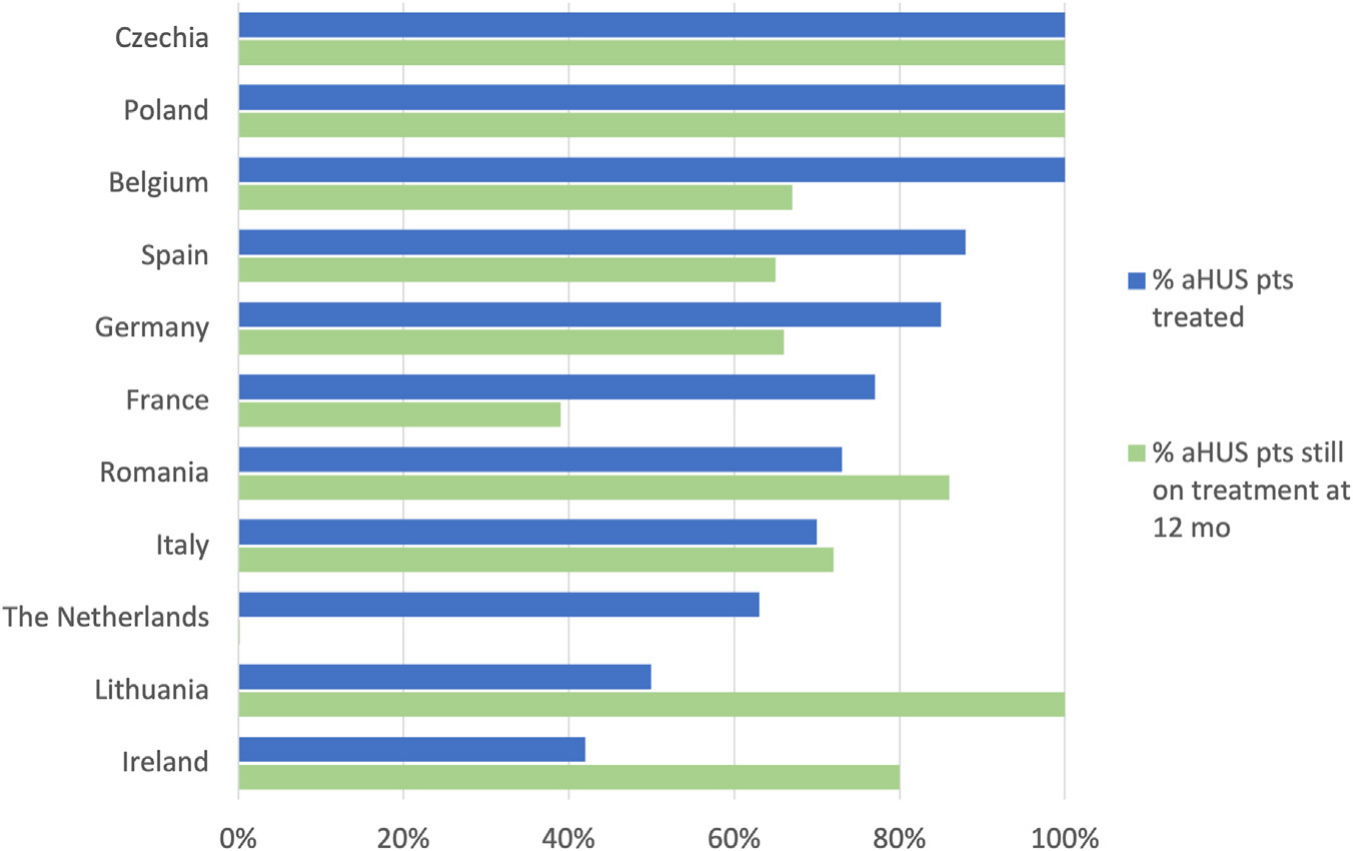
Utilisation of C5 inhibitors in patients with atypical haemolytic uraemic syndrome (aHUS) by country of residence. This figure illustrates the variation in C5 inhibitor use among patients with aHUS across different countries. Blue bars show the fraction of patients ever treated, and green bars the likelihood of remaining on treatment 12 months after initiation (based on Kaplan–Meier analysis). Czechia (n = 9), Poland (n = 9), Belgium (n = 6), Spain (n = 14), Germany (n = 34), France (n = 44), Romania (n = 8), Italy (n = 33), The Netherlands (n = 5), Lithuania (n = 4), and Ireland (n = 5). Abbreviations: %—percentage; aHUS—atypical haemolytic uraemic syndrome; pts—patients, mo—months.

Substantial differences in C5 inhibitor treatment practices between European countries were noted (p < 0.0001; Fig. 4). Treatment tended to be sustained longest in Eastern European countries (Czechia, Poland, Romania, Lithuania). Distinctly shorter treatment times were observed in the Netherlands and France, where 100% and 61% were discontinued within 12 months. Multivariable Cox regression analysis revealed that the observed country differences in C5 inhibitor treatment duration were not explained by the variation of case characteristics: using Germany as a reference, the adjusted likelihood of treatment discontinuation was significantly greater in the Netherlands (adjusted hazard ratio (aHR) 4.03; 95% confidence interval (CI) 1.14–11.13), France (aHR 2.13; 95% CI 1.21–3.85), and Ireland (aHR 8.26; 95% CI 2.65–21.56) independently of the globally reduced probability of discontinuation associated with genetic aHUS forms (aHR 0.35; 95% CI 0.21–0.56, reference: no cause identified aHUS) and an increasing likelihood of discontinuation with age at diagnosis (aHR 1.002; 95% CI 1.000–1.003), not shown. A diagnostic plot of the Cox regression's Martingale residuals vs. age at diagnosis indicated this predictor's linear relationship with the log event hazard. The assumption of proportional hazards was checked and not rejected on the Cox regression's scaled Schoenfeld residuals (Schoenfeld test for overall model: p = 0.10).

#### Treatment resumption

**Fig. 5:**
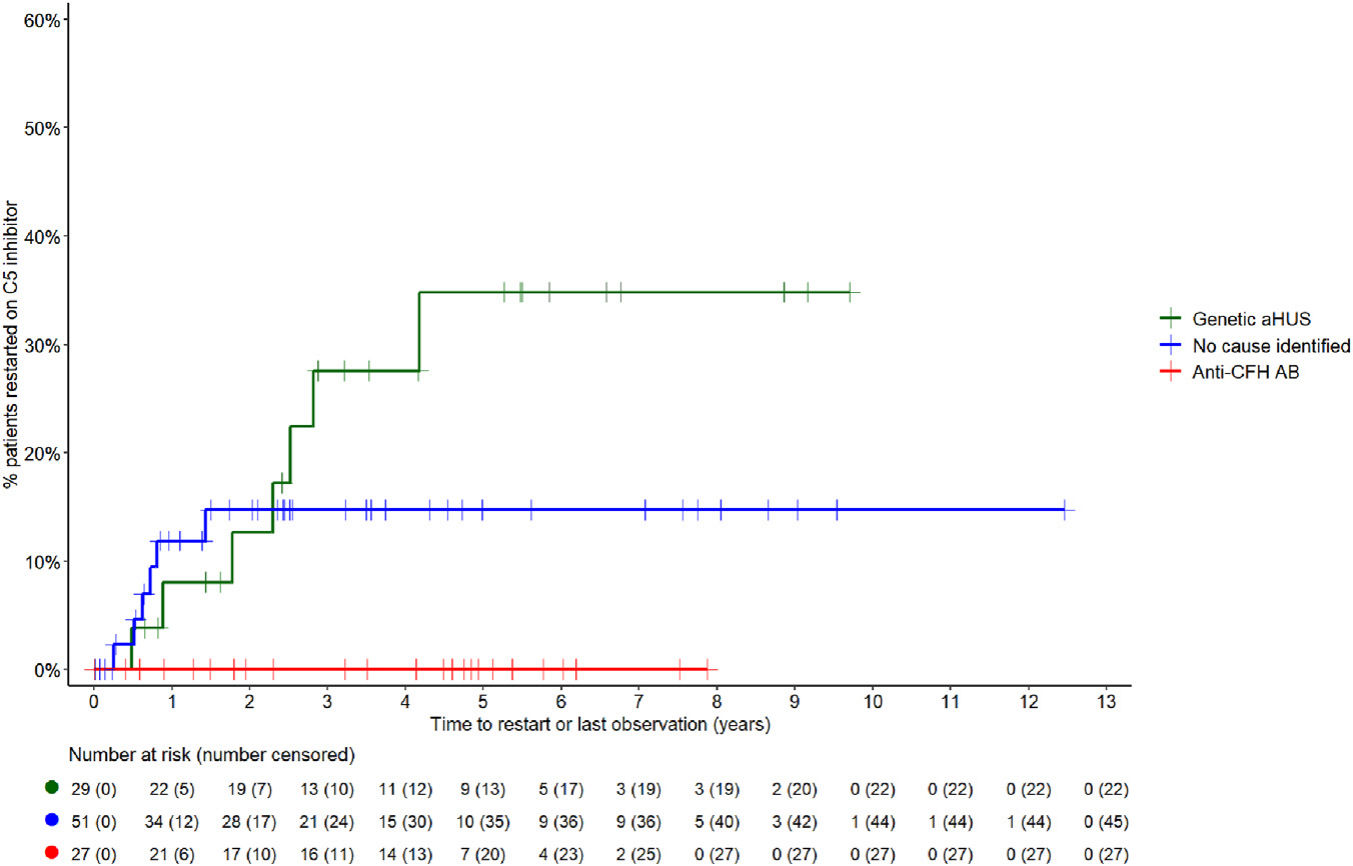
Time to treatment resumption following C5 inhibitor withdrawal in patients with atypical haemolytic uraemic syndrome (aHUS). This figure displays the time to treatment resumption after C5 inhibitor withdrawal, categorised by aHUS aetiology. Green indicates genetic aHUS, blue cases with no identified cause, and red anti-CFH antibody-mediated aHUS cases. Abbreviations: %—percentage; aHUS—atypical haemolytic uraemic syndrome; anti-CFH ab—anti-complement factor H antibody-mediated aHUS. Data availability: Time to treatment resumption data available for 107/107 aHUS patients.

Among the 107 patients initially treated with eculizumab in whom C5 inhibitor treatment was withdrawn, 94 (87.9%) remained off-therapy for a median (IQR) observation period of 3.5 (1.6–5.6) years, while 13 restarted treatment after a median (IQR) of 10 (6–28) months off-therapy. Among 13 patients who restarted treatment, eight had genetic disease forms (three mutations in C3 and CD46 each, one in CFI and one in CFH), three had no genetic test performed and in two genetic testing was negative. The overall long-term risk of re-starting treatment was 17.5% according to Kaplan–Meier analysis; the latest treatment resumption occurred 4.2 years after withdrawal. The risk of treatment resumption was 35% among patients with genetic, 15% in those with no cause identified and 0% in those with anti-CFH antibody-mediated aHUS (p = 0.047; Fig. 5). Among patients with genetic aHUS who discontinued treatment, relapses occurred in three out of 13 cases with CD46, one of three patients with C3, one of three patients with CFI and one of four patients with CFH mutations, whereas no relapses were observed in one patient with complement factor B (CFB) and one with a thrombomodulin (THBD) mutation after being off therapy for a mean (range) of 3.1 (0.8–5.3) years.

In 12 of the 13 patients, a detailed clinical and biochemical assessment was retrieved from the investigators. aHUS relapse was confirmed in 11 patients and strongly suspected in one. In the 11 patients with confirmed first relapse the mean lactate dehydrogenase (LDH) peak level was 850 U/L, the mean nadir platelet counts 87.000/μL and the mean estimated glomerular filtration rate (eGFR) nadir 36 ml/min per 1.73 m^2^. Two of the 13 patients required dialysis. At the last visit mean eGFR was 110 ml/min per 1.73 m^2^. On average, patients who restarted treatment required approximately four months for full clinical recovery and normalisation of laboratory values. Among the 13 patients in whom C5 inhibitor therapy was restarted after the first relapse, three patients were sustained on eculizumab, and one switched to ravulizumab (Fig. 1). Treatment was discontinued once again in nine patients. In six of these, eculizumab was reintroduced due to a second relapse while off-treatment. During the second relapse, the mean LDH peak level was 1100 U/L, the mean nadir platelet count was 63.000/μL and the mean eGFR nadir was 52 ml/min per 1.73 m^2^. None of the patients with a second relapse required dialysis. At the last visit mean eGFR was 122 ml/min per 1.73 m^2^. On average, patients with a second relapse required approximately two months for full clinical recovery and normalisation of laboratory values. In all six patients who experienced a second relapse, eculizumab was discontinued again. Three of these experienced a third relapse while off-treatment. Out of these, two had their treatment discontinued again, and one was sustained on eculizumab. One patient had a fourth relapse and was discontinued once more. In total, 99 of the 107 (92.5%) patients who discontinued treatment remained off therapy at the last observation. Among them, 24 patients with genetic aHUS were off therapy for a median (IQR) of 4.1 (2.2–5.6) years.

#### aHUS outcomes

Patient survival in the total aHUS cohort was >99%, with a single death recorded in a boy with genetic aHUS due to a CFH mutation. He underwent kidney transplantation four years after initiating C5 inhibitor therapy and passed away at 16 years of age from a post-transplant lymphoma.

**Fig. 6:**
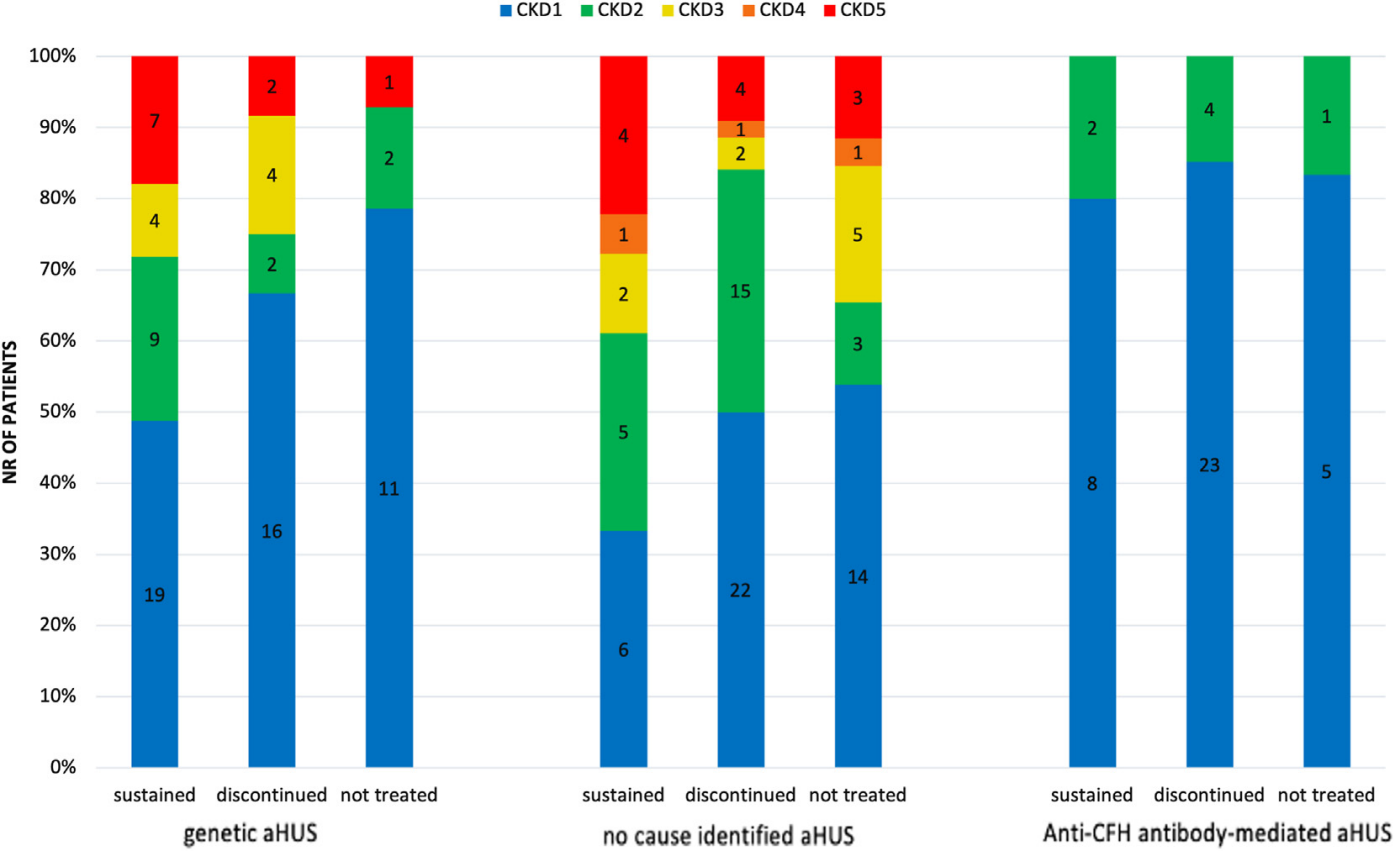
Kidney function outcomes by chronic kidney disease (CKD) stage in patients with atypical haemolytic uraemic syndrome (aHUS) followed for more than 3 months. Outcomes are stratified by aHUS type and C5 inhibitor treatment modality (sustained, discontinued, or never treated). This figure represents kidney function outcomes stratified by CKD stage, aHUS type, and C5 inhibitor treatment modality. Blue represents CKD stage 1, green stage 2, yellow stage 3, orange stage 4, and red stage 5. Abbreviations: Nr—number; %—percentage; CKD—chronic kidney disease; aHUS—atypical haemolytic uraemic syndrome; anti-CFH antibody—anti-complement factor H antibody-mediated aHUS. Data availability: 208/208 aHUS patients followed for more than 3 months.

For the 208 patients with aHUS followed for longer than three months (median (IQR) follow time 5.0 (2.5–7.6) years), the distribution of kidney function at last observation is summarised in Fig. 6 for patients on sustained C5 inhibitor therapy, those after treatment discontinuation and those who were never treated. The best overall outcomes were observed in anti-CFH antibody-mediated aHUS (no patients with CKD 3–5), whereas 23.4% (18/77) of patients with genetic and 26.1% (23/88) of no cause identified aHUS were in CKD 3–5 at last presentation. Global kidney outcomes did not differ significantly between patients treated with C5 inhibitors and those who were not, with CKD 3–5 occurring in 19.1% (31/162) and 21.7% (10/46), respectively. Among the treated patients, better outcomes were associated with C5 inhibitor withdrawal than with sustained therapy (CKD 3–5 in 13.7 (13/95) vs. 26.9% (18/67), p = 0.043).

At the last observation, one patient was in CKD 5 without kidney replacement therapy, six were on dialysis and 13 had undergone kidney transplantation. Out of the 20 patients who developed kidney failure, nine had genetic forms of aHUS (four CFH, three CFI, one CD46, and one C3 mutation) and 11 had no identified cause, including six without genetic testing and five with negative genetic screening results (not shown). Sixteen of the 20 patients with kidney failure had received a C5 inhibitor at any time following disease onset while four were never treated. Globally, kidney failure was present in 9.9% of patients who received C5 inhibitor therapy and in 8.7% of those who did not (not shown).

#### C5 inhibitor treatment in infectious (i)HUS

Among 472 patients diagnosed with iHUS, 87 (18.4%) received C5 inhibitor treatment, all with eculizumab (Fig. 1). These comprised 83 patients with STEC HUS and 4 with pneumococcal HUS. Median (IQR) treatment time was 9 (7–32) days. No patient received C5 inhibition for more than 12 months (Fig. 2). The use of eculizumab in patients with iHUS differed substantially by country: while in France 34% (49/143) received eculizumab, 18% (20/112) were treated in Italy, 17% (3/18) in Spain, 9% (7/79) in Germany and 12% (8/64) across Poland, Ireland, Belgium, Latvia, and Czechia, but none out of 56 cases reported in Slovenia, Austria, the Netherlands, Lithuania, and Greece (not shown).

Among 389 patients with iHUS followed for more than three months (median (IQR) follow time 3.1 (1.6–6.0) years), 25 (6.5%) developed kidney failure. The kidney failure rate was 9.5% and 5.8% among patients with and without C5 inhibitor therapy respectively (p = 0.24, not shown). One patient with STEC HUS, treated with eculizumab due to poor neurological status, died from brain oedema.

## Discussion

This study represents the most extensive real-world analysis of clinical practice patterns in patients with HUS since the introduction of pharmacological C5 inhibition. Our analysis was made possible by the systematic reporting of data with rare kidney diseases by the members of ERKNet to their core patient registry ERKReg and the nearly complete provision of additional information needed for this study by the sites, yielding a trans-national dataset with minimised selection and retention bias.

The study yielded several novel and partially unexpected findings. First, almost a quarter of patients diagnosed with aHUS after the marketing authorisation of eculizumab in the EU were never treated with C5 inhibitors, without any increase in use over time. Causes for withholding C5 inhibitors reported by the registry investigators included mild clinical presentation, successful first-line use of plasma exchange and restricted drug access, pointing to selective use of these very expensive drugs in European expert centres. Among aHUS patients with no identified cause, 60% did not undergo genetic testing, likely due to differences in access, institutional barriers, financial constraints, or earlier diagnostic practices. These patients likely represent a heterogeneous mix of aetiologies, including genetic aHUS, anti-CFH antibody-mediated aHUS, or iHUS, but reassigning them to specific categories was not feasible due to substantial gaps in diagnostic information. In addition, it is of note that female patients with aHUS were 36% less likely to be started on C5 inhibitor therapy than male patients. This disparity could be attributed to several factors. One possibility is that females might present with less severe forms of the disease. This has not been reported in the literature; aHUS outcomes did not differ between sexes in a large registry analysis of cases followed in the pre-eculizumab era.^8^ Alternatively, there could be a treatment decision bias in favour of males. Sex-based disparities in healthcare access have been reported in numerous areas of nephrology, e.g., women with CKD are less likely to receive antiproteinuric medication, female patients with kidney failure face greater barriers to be listed for transplantation and girls with kidney failure are less likely to receive pre-emptive transplantation than boys.^9^^,^^10^

Given the episodic nature of aHUS and the high cost of C5 inhibitor therapy, treatment discontinuation protocols have been proposed and applied.^3^^,^^11^ In this study, 50% of patients were discontinued within 2.4 years, with marked variation according to disease aetiology. While in 50% of anti-CFH antibody-mediated aHUS and no cause identified aHUS cases, C5 inhibition was terminated within 18 months and in 80% within 5–6 years, almost 60% of genetic aHUS cases were maintained on long-term therapy. Notably, significant variation in practice patterns was seen within the genetic group: whereas most patients with CFH and CFI mutations were sustained on therapy, two-thirds of patients with CD46 variants were discontinued within three years, reflecting the reportedly benign prognosis of aHUS related to the latter gene.^12^ However, several studies have demonstrated that both CFH and CD46 mutations are frequently linked to relapses.^3^^,^^13^ In addition, independently of the disease aetiology, the likelihood of treatment withdrawal increased with age at diagnosis. Finally, we observed major differences in C5 inhibitor treatment duration between European countries. These differences were independent of disease aetiology and patient age. The shortest treatment times were observed in the Netherlands and France, where early discontinuation policies have been evaluated in clinical studies.^3^^,^^13^^,^^14^ The most extensive treatment durations were observed in Eastern European countries, disproving the notion that national healthcare expenditure does not necessarily correlate to shorter treatment exposure.

While we estimated the global long-term risk of treatment resumption following discontinuation at 17.5%, patients with genetic form have more than twofold higher risk of disease recurrence than those without an identified aetiology. Disease recurrence was observed in patients with abnormalities in C3, CFI, CFH and, notably, in three of 13 patients with CD46 mutations. The recurrence risk associated with genetic forms may have been underestimated due to the limited follow-up time and the fact that three of the 13 recurrent patients had not undergone genetic testing. It is also of note that no episodes of disease recurrence were observed in any of the 27 patients with auto-CFH antibody-mediated aHUS in whom C5 inhibitor therapy was discontinued, suggesting that in this patient group treatment withdrawal is safe once the disease is effectively controlled by immunosuppressive therapy. Brocklebank et al. similarly demonstrated that relapse rates upon eculizumab withdrawal varied significantly by genetic background, with no relapses recorded in individuals without pathogenic genetic variants, supporting the observation that relapse is very unlikely in such cases.^15^

Probably thanks to high awareness of potential relapses, early detection, rapid access to care in expert centres and timely resumption of treatment, the clinical course of the TMA episodes was mostly mild; only two of the 13 patients transiently required dialysis and eGFR fully recovered in all patients. Consistent with these observations, Brocklebank et al. reported that among six individuals with functioning kidneys in whom eculizumab was restarted following withdrawal, none progressed to kidney failure, although one experienced a decline from CKD stage 2 to stage 3.^15^ In our study, the benign courses encouraged the treating clinicians to discontinue treatment again in nine of the 13 cases, all but one of whom remained off-treatment during subsequent observation. Hence, C5 inhibition was eventually weaned successfully in more than 92% of patients.

The impact of C5 inhibitor therapy on long-term kidney function outcomes could not be fully assessed in this registry study due to limited follow-up times and substantial bias by indication, whereby treatment was preferentially initiated and continued in patients with more severe initial disease manifestations. These inherent differences in baseline disease severity and other unmeasured factors make direct comparisons between treatment groups challenging. Furthermore, the better kidney outcomes observed in patients who discontinued C5 inhibitor therapy likely reflect a subset of individuals who demonstrated clinical improvement, making them suitable candidates for withdrawal. However, the inconsistent reporting of laboratory data, including complete blood counts and complement levels, limits the detailed assessment of disease activity and treatment response. Overall kidney function outcomes were quite satisfactory, with kidney failure occurring in as few as 11.7% of all cases. Outcomes were particularly favourable in anti-CFH antibody-mediated disease, where no CKD 4 or 5 cases were observed. Our results also confirm the long-term safety of C5 inhibitor discontinuation in aHUS.

C5 inhibitors were also found to be used in a surprisingly high fraction of patients with forms of iHUS. In many cases, C5 inhibitors may be administered as a precautionary measure in patients presenting with severe symptoms and/or yet unconfirmed diagnosis. Most patients with iHUS received only a single or a few doses. We observed major differences in C5 inhibitor utilisation between European countries. While the common use in France was largely explained by a national clinical trial performed during the observation period, the variation of usage in other countries may reflect differences in national regulations and policies regarding off-label drug use. The efficacy of C5 inhibition in iHUS is controversial and difficult to evaluate even in controlled trials due to treatment bias favouring uncontrolled rescue administration in the most severely affected children, who are prone towards poor outcomes.^4^, ^5^, ^6^ This bias was also evident in our analysis, as patients with iHUS who received eculizumab had worse kidney function at the last observation.

In conclusion, our comprehensive real-world analysis of HUS treatment practices and outcomes revealed that the concept of C5 inhibition, while being the standard of care in aHUS, is applied in less than 80% of newly diagnosed patients in European specialised centres. Discontinuation practices vary widely, ranging from rapid withdrawal in all patients to selective, variably timed discontinuation based on presumed risk profiles. Patients with genetic disease forms are more frequently maintained on therapy and are at increased risk of disease recurrence when discontinued. The overall treatment experience with the current practice patterns is favourable, with only 12% of patients developing permanent kidney failure despite C5 inhibitor therapy and no increased risk of poor long-term kidney function outcomes in patients in whom treatment is electively discontinued. The best long-term outcomes are observed for anti-CFH antibody-mediated aHUS, with no relapses after treatment and a very low risk of permanent kidney damage.

## Contributors

AV was responsible for conceptualisation, study design, data collection, data curation, formal analysis, data interpretation, writing the original draft, reviewing, and editing the manuscript, visualisation, project administration, and supervision. ALSL, MCM, OB, AA, AG, SL, MF, AJ, GA, NK, EV, MVD, TKL, NS, and SD contributed to data collection, updating, and entering data into the registry, project administration, data curation, reviewing the manuscript, providing suggestions for revisions, and editing the final draft. JH supported statistical analysis, data analysis, data collection, and contributed to reviewing and editing the manuscript, and had direct access to the data. MV, SS, and NCAJVDK were involved in data collection, supervision, manuscript review, and editing. FS contributed to conceptualisation, study design, data curation, formal analysis, data interpretation, data visualisation, writing the original draft, reviewing, and editing the manuscript, project administration, and supervision. More than one author, including AV, FS, and JH, directly accessed and verified the underlying data reported in the manuscript. All authors have reviewed and approved the final manuscript and agree to be accountable for all aspects of the work, ensuring the accuracy and integrity of the data and analysis.

## Data sharing statement

The data for this study, including deidentified participant data, is available through the European Rare Kidney Disease Registry (ERKReg) upon approval of a proposal to the ERKNet board. ERKReg's comprehensive data access policy ensures high standards of security, privacy, and integrity. Researchers can request access based on role and need, following a formal review process. Please review the policy and submit requests here: www.erknet.org/patients-registry/data-access-requests. For questions, contact erkreg@erknet.org.

## Declaration of interests

All authors declare the following potential conflicts of interest related to the content of this manuscript: MCM received speaker honoraria and a travel grant from Alexion. OB received speaker honoraria from Alexion and Samsung. AA served on the Data Monitoring Committee for the UK trial on C5 inhibitor discontinuation. MF received speaker honoraria, payment for expert testimony, consultancy fees, and a travel grant from Alexion. GA received speaker honoraria and travel grants from Alexion (AstraZeneca Rare Diseases) and served on its Board. JH works as a statistician at ERKNet, which received payments from Vertex, Novartis, and Sobi for research collaborations. JH performed statistical analyses and created project reports on behalf of ERKNet: “Analysis of the ERKReg database for ADPKD & AMKD studies” for Vertex, “Disease characteristics and treatment patterns of C3G and IC-MPGN in Europe: a multicentre retrospective analysis using the ERKReg database” for Novartis, and “A C3G and Primary IC-MPGN Retrospective Observational Study - Fit-For-Purpose Summary Report” for Sobi. MV received consultancy fees from Novartis, BioCryst, Roche, Apellis, speaker honoraria from Roche, Novartis, Alexion, Vifor, and Travere, and a grant from the Italian Ministry of Health to her institution. NCAJVDK received grants from Apellis and Novartis as principal or sub-investigator for international trials on pegcetacoplan and iptacopan for C3G treatment, consulting fees from Samsung, Alexion, and Novartis, speaker fees from Novartis and Sobi, travel grants from Samsung and Sobi, and served on a board for Roche, with all payments made to her institution. FS received consulting fees from Samsung Bioepis for participation in Scientific Advisory Board meetings, with payment made to him, and from Alexion for consulting on paediatric trial programs in potential new indications for C5 inhibitors while participating in the sAlexion Global aHUS Registry Steering Committee, with payments made to his institution. The remaining authors declare no conflicts of interest. No other relevant affiliations or financial involvements exist beyond those disclosed.

## Appendix ASupplementary data

Supplementary Materials

ERKNet TMA Working Group
