# Supplementary material for: Real-world use of complement inhibitors for haemolytic uraemic syndrome: an analysis of the European Rare Kidney Disease Registry cohort
Source: eClinicalMedicine. 2025 Mar 27;82:103159. doi: 10.1016/j.eclinm.2025.103159 (PMC11987679; doi:10.1016/j.eclinm.2025.103159)
Supplement: Supplementary Materials [file mmc1.pdf]

Supplementary Material for the Article

Real-world use of complement inhibitor for haemolytic uraemic syndrome in clinical practice: a retrospective analysis of the European Rare Kidney Disease Registry cohort

eClinicalMedicine

Table of Contents

1. Study Protocol

- Introduction
- Methods and Analysis
- Ethics and Dissemination
- Funding Statement
- Conclusion
- Declaration of interests

2. List of Collaborators

1. Study protocol

Introduction

A study protocol is a critical document that details the methodology of a research project, enabling team members to follow standardized procedures and ensuring reproducibility. This protocol outlines a retrospective cohort study on Haemolytic Uremic Syndrome (HUS) conducted within the European Rare Kidney Disease Registry (ERKReg). The study aims to evaluate clinical outcomes, treatment patterns, and variations in diagnostic approaches for patients diagnosed with HUS.

HUS is a rare, life-threatening condition characterised by thrombocytopenia, microangiopathic haemolytic anaemia, and acute kidney injury. Despite the availability of complement inhibitors, significant variability in their use exists across Europe. This study seeks to address gaps in understanding these variations, focusing on long-term outcomes and treatment discontinuation practices.

Methods and Analysis

Study Design

This retrospective cohort study analysed longitudinal data collected between November 2018 and January 2024. The registry captures real-world variability in diagnostic and treatment practices across 24 European countries.

Study Population

Inclusion Criteria:

- Patients diagnosed with HUS (aHUS or iHUS) after 2011 or before 2011 if treated with eculizumab in a clinical trial.

- Availability of complement inhibitor treatment information.

Exclusion Criteria:

- Lack of complement inhibitor treatment information.

- Non-complement-mediated HUS forms (e.g., Cobalamin C deficiency, DGKE nephropathy).

- HUS manifesting post-kidney transplantation.

- Inconsistent data in iHUS cases.

- Patients receiving prophylactic complement inhibitors around kidney transplantation.

Cohort Details:

The study cohort included 710 patients (238 with aHUS and 472 with iHUS):

- aHUS subgroup: Anti-CFH antibody-mediated (n = 48), genetic cause identified (n = 85), no identified cause (n = 105)

- iHUS subgroup: Shiga toxin-producing Escherichia coli (STEC) HUS (n = 456),

- Pneumococcal HUS (n = 16)

Data Collection

The registry collects the following data:

- Demographic information: age, sex, disease onset.

- Diagnostic findings: genetic testing results, antibody assays.

- Treatment details: plasma exchange, dialysis, kidney transplantation, and complement inhibitor use (including discontinuation status).

- Kidney function: serum creatinine levels recorded at registry enrolment and updated annually.

- Focused queries: reasons for complement inhibitor non-use and post-discontinuation thrombotic microangiopathy (TMA) episodes.

Statistical Analysis

Summary Statistics: Categorical variables were presented as counts and percentages; continuous variables as means (SD) or medians (IQR).

Group Comparisons:

- Mann-Whitney U test for non-normal distributions.

- Kruskal-Wallis test for >2 groups with non-normal or unequal variances.

- Chi-squared/Fisher's exact tests for categorical variables.

- Survival Analysis: Kaplan-Meier methods were used for treatment duration.

- Multivariable Cox Regression: Hazard ratios (HR) were estimated to identify factors influencing treatment discontinuation. The Cox proportional hazards model assumptions (proportional hazards and linearity of covariates) were validated using graphical diagnostics based on Schoenfeld and Martingale residuals.

Sample Size Estimation

Due to few to no published evidence on effect sizes in the aHUS and iHUS populations for the variable associations and/or differences tested by our study, we did not conduct any a-priori power analyses and understood our primary goal in exploring and primarily quantifying these relationships based on the available data in ERKReg.

Handling of Missing Data

All analyses were conducted using available data, with no imputation or estimation of missing values.

Ethics and Dissemination

Ethical Considerations

Ethical approval was obtained from local ethics committees at all participating centres. Informed consent was secured from all patients or their legal representatives before registry enrolment.

Confidentiality and Data Ownership

Personal data were collected, stored, and maintained according to the General Data Protection Regulation (GDPR). Data ownership and storage policies comply with ERKNet guidelines.

Dissemination

Study results will be disseminated through peer-reviewed publication and presentations at relevant scientific conferences. Participants will be informed of key findings where appropriate.

Funding Statement

This research was supported by the European Reference Network for Rare Kidney Diseases (ERKNet). ERKNet is funded by the European Union within the framework of the “EU4Health Programme 2021-2027”. The funding body had no role in the study design, data collection, analysis, or interpretation, nor in the writing of the report or the decision to submit this paper for publication.

Conclusion

This protocol describes a retrospective cohort study aimed at addressing gaps in understanding treatment patterns, diagnostic variability, and long-term outcomes in HUS. The findings will

contribute to the standardization of treatment practices and improved patient outcomes across Europe.

Declaration of interests

All authors declare the following potential conflicts of interest related to the content of this manuscript: MCM received speaker honoraria and travel grant from Alexion. OB received speaker honoraria from Alexion and Samsung. AA served on the Data Monitoring Committee for the UK trial on C5 inhibitor discontinuation. FM received consultancy fees, speaker honoraria and travel grant from Alexion. GA received speaker honoraria, travel grants, and/or consulting fees from Alexion (AstraZeneca Rare Diseases), and collaborated with Recordati Rare Diseases, Advicenne, Chiesi, Alnylam, Kyowa Kirin, Dicerna, and Novo Nordisk. JH's institution received grants from Vertex, Novartis, and Sobi. MV received consultancy fees from Novartis, BioCryst, Roche, Apellis and speaker honoraria from Roche, Novartis, Alexion, Vifor and Travere, and a grant from the Italian Ministry of Health to their institution. NVDK received grants from Apellis and Novartis, consulting fees from Samsung, Alexion, and Novartis, speakers' fees from Novartis and Sobi, and travel grants from Samsung and Sobi, and served on a board for Roche. FS received consultancy fees and research funding from Alexion, Roche and Samsung, and participated in the Alexion Global aHUS Registry Steering Committee. The remaining authors declare no conflicts of interest. No other relevant affiliations or financial involvements exist beyond those disclosed.

2. List of Collaborators (ERKReg investigators contributing patients to this study):

Austria: Klaus Arbeiter, Alexandra Potemkina (Vienna). Belgium: Detlef Böckenhauer, Djalila Mekahli, Maria Van Dyck, Albert Herelixka, Kathleen Claes (Leuven); Ann Raes, Evelien Snauwaert, Johan Vande Walle (Ghent). Croatia: Lovro Lamot (Zagreb); Czechia: Jakub Zieg, Naděžda Šimánková, Alena Parikova (Prague). Estonia: Mai Rosenberg (Tartu). France: Jerome Harambat, Lise Allard (Bordeaux); Justine Bacchetta, Anne-Laure Sellier-Leclerc, Lisa Condamine, Dounia Habchi, Lydia Slimani, Amina Talhi, Sacha Flammier (Lyon); Denis Morin, Marc Fila, Julie Tenenbaum, Katja Zurbonsen, Moglie La Quintrec (Montpellier); Laurence Heidet, Olivia Boyer, Kahina Saidoun; Bertrand Knebelmann, Aude Servais, Josephine Cornet, Yann Nedelec (Paris Necker); Stephane Decramer, Nabila Moussaoui, Aurelie Rigal; Dominique Chauveau, David Ribes (Toulouse). Germany: Anja Büscher, Tanja Kier (Essen); Jun Oh, Sebastian Loos, Madelaine Wingerath (Hamburg); Marcus Weitz, Kathrin Buder, Lena Lechler (Tübingen); Franz Schaefer, Susanne Schaefer (Heidelberg); Dieter Haffner, Nele Kanzelmeyer, Lena Brunkhorst, Jens Drube (Hannover); Sabine König, Sabine Kollmann (Münster). Greece: Nikoleta Printza (Thessaloniki). Ireland: Peter J Conlon, Aamir Elhassan Elhussein (Dublin, Beaumont Hospital), Atif Awan, Michael Riordan, Shirley Bracken (Dublin, CHI). Italy: Piero Ruggenenti, Valentina Fanny Leone, Amantia Imeraj (Bergamo); Paola Romagnani, Francesca Becherucci, Luigi Cirillo (Florence); Enrico Verrina, Francesca Lugani (Genoa); Giovanni Montini, Gianluigi Ardissino, Valentina Capone, Maria Cristina Mancuso (Milan); Gabriele Malgieri (Naples Santobono-Pausilipon), Francesco Trepiccione, Miriam Zacchia (Naples-UOC); Enrico Vidal, Germana Longo, Mattia Parolin, Nicola Bertazza Partigiani (Padua); Francesco Emma, Marina Vivarelli, Antonio Gargiulo, Chiara Bettini (Rome, Ospedale Bambino Gesu); Giuseppe Grandaliano, Rocco Baccaro, Daniela Palazzetti (Rome, Ospedale Gemelli); Dario Roccatello, Savino Sciascia, Giulio del Vecchio (Ospedale HUB Torino Nord); Licia Peruzzi, Vitor Hugo Martins (Turin, Regina Margherita Hospital). Latvia: Ilze Andersone, Sandra Derkevica (Riga). Lithuania: Augustina Jankauskiene, Rimante Cerkauskiene, Renata Vitkevic, Marius Miglinas (Vilnius). Poland: Aleksandra Zurowska,

Magdalena Drozynska-Duklas (Gdansk); Marcin Tkaczyk, Małgorzata Buśko, Anna Krakowska (Lodz). Portugal: Joaquim Calado (Lisbon). Romania: Adrian Catalin Lungu, Anca Marin, Valentin Mocanu (Bucharest). Slovenia: Tanja Kersnik Levart, Aleksandra Vujović (Ljubljana); Nataša Marčun Varda (Maribor). Serbia: Dušan Paripović (Belgrade). Spain: Gema Ariceta, Victor Beltran Perez, Carla Soto (Barcelona); Mar Espino Hernandez, Enrique Morales, Teresa Cavero, Hernando Trujillo (Madrid). The Netherlands: Antonia Bouts (Amsterdam); Elisabeth Cornelissen, Nicole van de Kar, Linda Koster-Kamphuis, Tom Nijenhuis (Nijmegen).
