# Supplementary material for: Real-world use of complement inhibitors for haemolytic uraemic syndrome: an analysis of the European Rare Kidney Disease Registry cohort
Source: eClinicalMedicine. 2025 Mar 27;82:103159. doi: 10.1016/j.eclinm.2025.103159 (PMC11987679; doi:10.1016/j.eclinm.2025.103159)
Supplement: ERKNet TMA Working Group [file mmc2.docx]

# Study Group Members for PubMed Indexing

| First and Middle Names | Surname |
| --- | --- |
| Nicole C.A.J | van de Kar |
| Marina | Vivarelli |
| Savino | Sciascia |
| David | Kavanagh |
| René | Andersen |
| Mia | Faerch |
| Soren | Rittig |
| Miquel | Blasco |
| Pedro Arango | Sancho |
| Alvaro | Madrid |
| Gema | Ariceta |
| Loreto | Gesualdo |
| Camillo | Carrara |
| Piero | Ruggenenti |
| Kai-Uwe | Eckardt |
| Jan | Halbritter |
| Dominik | Müller |
| Adrian | Schreiber |
| Evelyn | Seelow |
| Yahsou | Delmas |
| Jerome | Harambat |
| Brigitte | Llanas |
| Nathalie | Godefroid |
| Eric | Goffin |
| Johann | Morelle |
| Adrian Catalin | Lungu |
| George | Reusz |
| Péter | Sallay |
| Attila | Szabo |
| Kalman | Tory |
| Jan Ulrich | Becker |
| Kathrin | Burgmaier |
| Volker | Burst |
| Sandra | Habbig |
| Max | Liebau |
| Roman-Ulrich | Mueller |
| Lutz | Weber |
| Mette | Damholt |
| Anne-Lise | Kamper |
| Karl Emil | Nelveg-Kristensen |
| Hanne | Nørgaard |
| Ida Maria | Schmidt |
| Soeren | Soerensen |
| Wladimir | Szpirt |
| Agnieszka | Jaskólska |
| Monika | Miklaszewska |
| Anna | Moczulska |
| Elżbieta | Szczęsny-Choruz |
| Katarzyna | Zachwieja |
| Peter | Conlon |
| Atif | Awan |
| Michael | Wiesener |
| Anja | Büscher |
| Rainer | Büscher |
| Lars | Pape |
| Francesca | Becherucci |
| Paola | Romagnani |
| Ann | Raes |
| Thomas | Renson |
| Evelien | Snauwaert |
| Johan | Vande Walle |
| Jill | Vanmassenhove |
| Mark | Eijgelsheim |
| Casper | Franssen |
| Coen | Stegeman |
| Florian | Grahammer |
| Thomas | Henne |
| Tobias | Huber |
| Christian | Krebs |
| Sebastian | Loos |
| Anne | Mühlig |
| Jun | Oh |
| Ulf | Panzer |
| Raphael | Schild |
| Jessica | Kaufeld |
| Stefanie | Haeberle |
| Franz | Schaefer |
| Tanja | Wlodkowski |
| Juuso | Tainio |
| Elisa | Ylinen |
| Obbo | Bredewold |
| Wieneke | Michels |
| Dorien | Peters |
| Antonius | Rabelink |
| Arghya | Ray |
| Joris | Rotmans |
| Siebe | Spijker |
| Y.K. Onno | Teng |
| Kathleen | Claes |
| Noel | Knops |
| Tanja | Kersnik Levart |
| Anamarija | Meglič |
| Gregor | Novljan |
| Agnieszka | Gach |
| Monika | Pawlak-Bratkowska |
| Małgorzata | Stańczyk |
| Marcin | Tkaczyk |
| Pierre | Cochat |
| Anne-Laure | Sellier-Leclerc |
| Teresa | Cavero |
| Eduardo | Guiterrez |
| Joaquin | Martinez |
| Enrique | Morales |
| Hernando | Trujillo |
| Gianluigi | Ardissino |
| Valentina | Capone |
| Sara | Testa |
| Denis | Morin |
| Martin | Konrad |
| Ilaria | Luongo |
| Gabriele | Malgieri |
| Luigi Annicchiarico | Petruzzelli |
| Wilbert | van der Meijden |
| Jack | Wetzels |
| Mattia | Parolin |
| Enrico | Vidal |
| Thérésa | Kwon |
| Olivia | Boyer |
| Aude | Servais |
| Laurent | Mesnard |
| Alena | Parikova |
| Silvie | Rajnochova-Bloudickova |
| Janka | Slatinska |
| Ondrej | Viklicky |
| Martin | Bezdicka |
| Nadezda | Simankova |
| Dana | Thomasova |
| Jakub | Zieg |
| Francesco | Emma |
| Rocco | Baccaro |
| Giuseppe | Grandaliano |
| Alessandro | Naticchia |
| Francesco | Pesce |
| Margherita | Baldassarri |
| Andrea | Guarnieri |
| Anna Maria | Pinto |
| Alessandra | Renieri |
| Stephane | Decramer |
| Stanislas | Faguer |
| David | Ribes |
| Thomas | Simon |
| Stephanie | Tellier |
| Daniel | Gale |
| Christoph | Licht |
| Michal | Malina |
| Candice | Roufosse |
| Neil | Sheerin |
| Susana Carvajal | Arjona |
| Renée | de Wildt |
| Uwe | Korst |
| Christiane | Mockenhaupt |
| Francisco | Monfort |
| Mireya Vicenta Rios | Carratala |
